# Comorbidity of eosinophilic chronic rhinosinusitis in chronic eosinophilic pneumonia

**DOI:** 10.1002/rcr2.1236

**Published:** 2023-10-16

**Authors:** Hiroatsu Hatsukawa, Masaaki Ishikawa, Tomoyuki Hirai, Kazuo Endo, Emiko Saito, Hirotaka Matsumoto, Kouya Okazaki

**Affiliations:** ^1^ Department of Otolaryngology, Head and Neck Surgery Hyogo Prefectural Amagasaki General Medical Center Amagasaki Japan; ^2^ Department of Respiratory Medicine Hyogo Prefectural Amagasaki General Medical Center Amagasaki Japan

**Keywords:** chronic eosinophilic pneumonia, eosinophilic chronic rhinosinusitis, nasal polyps; hypereosinophilia, sinonasal computed tomography

## Abstract

We aimed to elucidate details of comorbid chronic rhinosinusitis (CRS) in chronic eosinophilic pneumonia (CEP) under the collaboration between otolaryngologists and pulmonologists in a prospective study. The CEP diagnosis was performed by pulmonologists based on clinical symptoms, laboratory findings, and/or eosinophilia detected in bronchoalveolar lavage. All patients were referred to otolaryngologists before undergoing oral corticosteroid treatment for CEP. Ten CEP cases visited to otolaryngologists. All cases showed bilateral sinonasal inflammation in computed tomography (CT), indicating comorbid CRS. Nasal polyps (NPs) were observed in 50% of patients on endoscopy. Eighty percent of patients were diagnosed with eosinophilic CRS. In blood eosinophil levels and the mucosal eosinophil count, there were no significant differences between CRS without and with NPs. In Lund–Mackay CT total scores, among‐individual variability was observed in CRS with NPs. The collaboration revealed blood/sinonasal eosinophilia and the variability in Lund–Mackay CT total scores as remarkable findings about the comorbid CRS.

## Graphical abstract

The comorbidity of eosinophilic chronic rhinosinusitis in chronic eosinophilic pneumonia can occur.

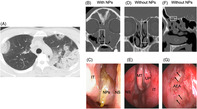

## INTRODUCTION

Chronic eosinophilic pneumonia (CEP) is an idiopathic pulmonary disease characterized by abnormal infiltrations in the lungs.^1^ Prolonged respiratory symptoms, such as cough (60–90%) or shortness of breath/dyspnea (20%–50%), are common. Some patients have either a prior history or concurrent development of allergic diseases, such as atopy, asthma, and allergic rhinitis. Furthermore, approximately 10% of CEP patients present with nasal polyps (NPs)^2^, ^3^; this finding is one of the representative phenotypes in chronic rhinosinusitis (CRS).^4^ To date, detailed phenotypes excluding NPs have not been reported for CRS with CEP. In this study, we aimed to prospectively investigate the potential of comorbid CRS at the onset of CEP and its phenotypes. For this purpose, otolaryngologists collaborated with pulmonologists.

## METHODS

The study was conducted between November 1, 2021 and December 31, 2022. The Research Ethics Committee in our institution approved the study protocol (approval no. 3‐18). When patients visited pulmonologists for pneumonia with blood eosinophilia showing an absolute eosinophil count (AEC) of ≥500/μL,^5^ the pulmonologists consulted otolaryngologists for potential CRS comorbidities before beginning oral corticosteroid treatment. CEP diagnosis was performed by pulmonologists based on the clinical symptoms lasting 2 weeks, abnormal chest findings in computed tomography (CT), exclusion of other known eosinophilic pneumonia such as allergic bronchopulmonary aspergillosis and eosinophilic granulomatosis with polyangiitis (EGPA), and/or eosinophilia detected in bronchoalveolar lavage (BAL) (over 25%).^1^ Radiologists checked the sinonasal and chest CT findings of all patients, and then commented its findings. In sinonasal CT, a Lund‐Mackay CT total score ranging from 0 to 24 was used.^6^ When sinonasal CT findings indicated the comorbid CRS with pneumonia, otolaryngologists and pulmonologists performed full‐house endoscopic sinus surgery and BAL under general anaesthesia, respectively. To assess CRS phenotypes, otolaryngologists collected NPs located in the middle meatus in CRS with NPs or edematous mucosa located in the ethmoid cavity in CRS without NPs. Prior to these procedures, informed consent was obtained from all patients to obtain sinonasal samples and explore the CRS phenotypes.

Eosinophilic CRS was diagnosed based on the Japanese Epidemiological Survey of Refractory Eosinophilic Chronic Rhinosinusitis (JESREC) scoring system.^7^ The system consists of clinical scores, including disease side (both sides, 3 points), existence of NPs (2 points), CT shadow (ethmoidal ≥ maxillary, 2 points), blood eosinophilic percentage (>2% and ≤5%—4 points; > 5% and ≤10%—8 points; >10%—10 points). When the total score was ≥11 and with a mean mucosal eosinophil count in the three densest areas with cellular infiltrate beneath the epithelial surface ≥70/high‐power filed (HPF: magnification, ×400),^7^ the patient was diagnosed with eosinophilic CRS. The Mann–Whitney *U* test was used for the association between NPs and eosinophil data/Lund‐Mackay CT score. A 5% significance level was considered as a statistically significant difference. R software version 3.6.1. was used for all statistical analyses.

## RESULTS

**FIGURE 1:**
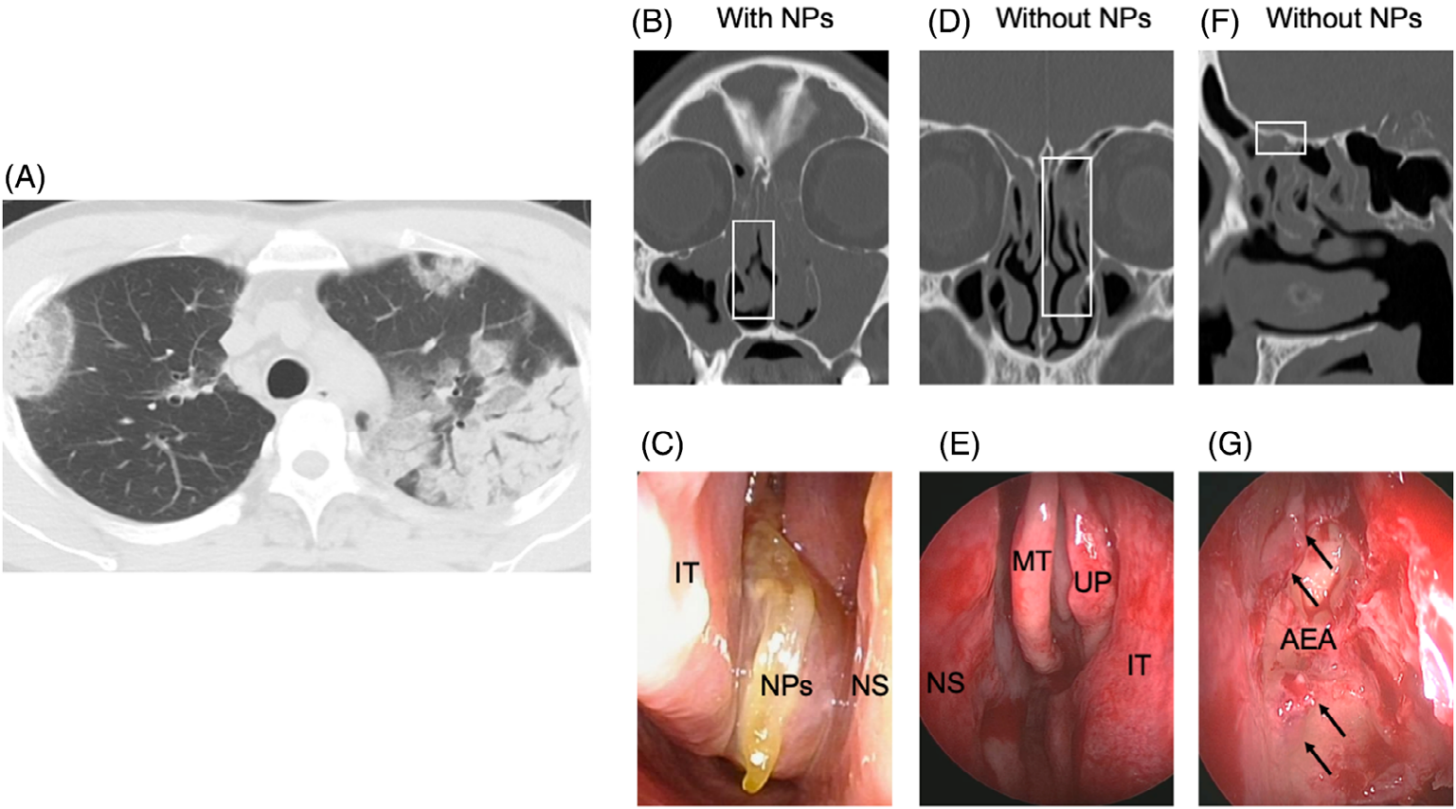
Representative imaging findings of comorbid CRS at onset of CEP. Representative chest CT findings in ID 1 (A). Representative findings of comorbid CRS with (B, C) and without NPs (D–G) in CEP: Sinonasal CT findings (B, D, and F); Endoscopic findings (C, E, and G). The figures B and C were in ID 1, while figures D, E, F, and G in ID 5. Black arrows indicate the edematous mucosa in the ethmoidal tissues. Endoscopic findings correspond with the squares in the CT findings. AEA, anterior ethmoidal artery; CEP, chronic eosinophilic pneumonia; CRS, chronic rhinosinusitis; CT, computed tomography; IT, inferior turbinate; MT, middle turbinate; NPs, nasal polyps; NS, nasal septum; UP, uncinate process.

Ten patients were referred to otolaryngologists and underwent CT, endoscopic nasal examination, and screening for blood eosinophil count. CT revealed pneumonia (Figure 1A) and bilateral sinonasal inflammation (Figure 1B,D) in all patients.

The clinical characteristics of the patients are shown in Table 1. In CEP diagnosis, five patients (ID 5, 6, 8, 9, and 10) showed eosinophilia detected in the BAL. The other patients did not undergo the BAL, being diagnosed with CEP based on the clinical findings including blood eosinophilia. In terms of medication, 40% of the patients received inhaled corticosteroids to control concurrent asthma (ID 3, 7, 8, and 9), while none received oral corticosteroid treatment. Seven patients had been suffered from CRS symptoms prior to the CEP onset, being treated with antihistamines/steroid nasal spray (ID 1, 2, 3, 6, 7, 8 and 9). Seven patients were diagnosed with comorbid allergic rhinitis (ID 1, 2, 3, 5, 6, 8, and 9). The percentages of current‐, past‐, and non‐smoker were 10%, 50%, 40%, respectively. Nasal endoscopy revealed NPs in 50% of the patients (ID 1, 2, 3, 4, and 9). For instance, case 1 of CRS with NPs showed severe inflammation on sinonasal CT, and congested NPs in the common nasal meatus (Figure 1B,C). The remaining 50% (ID 5, 6, 7, 8, and 10) did not show NPs (Figure 1E); however, inflammation of the anterior and posterior ethmoids was observed on CT (Figure 1D,F). In these cases, otolaryngologists confirmed the edematous mucosa of the ethmoid cavity (Figure 1G). In CT findings, 70% of patients showed mild sinonasal inflammation ranging from 4 to 6 in the Lund‐Mackay CT score (ID 4, 5, 6, 7,8, 9, and 10), while 30% severe one ranging from 18 to 24 (ID 1, 2, and 3). In the sub‐scales of the Lund‐Mackay CT score, all cases showed higher scores in the ethmoid sinus than ones in the maxillary sinus. The three cases ranging from 18 to 24 in the Lund‐Mackay CT total score showed severe inflammation in not only ethmoid sinus but also ostiomeatal complex (ID 1, 2, and 3), while the others ranging from 4 to 6 showed mild inflammation mainly in ethmoidal sinus (ID 4, 5, 6, 7,8, 9, and 10).

**TABLE 1:** Clinical characteristics of comorbid chronic rhinosinusitis at the chronic eosinophilic pneumonia onset.

ID	Age, sex	Asthma status/treatment	NPs	1. CRS symptoms prior to CEP onset	Lund–Mackay CT score				Eosinophilic data			
									Blood			
					Total score	Anterior/Posterior ethmoid sinus	Maxillary sinus	Frontal sinus/sphenoid sinus/ostiomeatal complex	%	AEC (/μL)	Sinonasal tissues (/HPF)	BAL (%)
				2. Allergic rhinitis								
				3. Smoking status								
1	43, M	−	+	1:(+), 2: (+), 3: Current‐smoker	18	3/4	3	0/4/4	51.0	8517	317	−
2	46, M	−	+	1:(+), 2: (+), 3: Past‐smoker	24	4/4	4	4/4/4	40.8	6038	116	−
3	71, F	+/ICS^a^	+	1:(+), 2: (+), 3: Non‐smoker	19	3/4	2	4/2/4	39.0	2535	1000	−
4	35, M	−	+	1:(−), 2: (−), 3: Non‐smoker	4	2/2	0	0/0/0	38.6	4130	85	−
5	41, M	−	−	1:(−), 2: (+), 3: Past‐smoker	4	2/2	0	0/0/0	44.9	5568	266	89
6	39, F	−	−	1:(+), 2: (+), 3: Non‐smoker	4	2/2	0	0/0/0	76.0	20,748	663	94
7	63, F	+/ICS^b^	−	1:(+), 2: (−) 3: Past‐smoker	4	2/2	0	0/0/0	58.4	6249	70	−
8	22, M	+/ICS^b^	−	1:(+), 2: (+), 3: Non‐smoker	6	2/2	2	0/0/0	51.0	5610	120	96
9	55, M	+/ICS^a^	+	1:(+), 2: (+), 3: Past‐smoker	4	2/2	0	0/0/0	15.1	966	35	33
10	65, M	−	−	1:(−), 2: (−), 3: Past‐smoker	6	3/3	0	0/0/0	14.2	1761	1	31

*Note*: The JESREC scoring system7 was used for eosinophilic CRS diagnosis. All patients showed bilateral CRS in computed tomography and eosinophil percentage of >10% in the blood, with total scores ≥11 in the JESREC scoring system.

Abbreviations: AEC, absolute eosinophil count; BAL, bronchoalveolar lavage; CRS, chronic rhinosinusitis; CT, computed tomography; HPF; high‐power field; ICS, inhaled corticosteroid; JESREC, Japanese Epidemiological Survey of Refractory Eosinophilic Chronic Rhinosinusitis scoring system; NPs, nasal polyps.

^a^ 250 μg/day of ICS dose.

^b^ 500 μg/day of ICS dose.

All patients exhibited bilateral CRS and eosinophil percentage of >10% in the blood, with total scores ≥11 in the JESREC scoring system. In addition, hypereosinophila (AEC of ≥1500/μL)^5^ was observed in 90% of the patients. Eight patients had the mucosal eosinophil count of ≥70/HPF, being diagnosed with eosinophilic CRS (ID 1–8). In the remaining two patients diagnosed with non‐eosinophilic CRS (ID 9 and 10), the mean numbers of mucosal eosinophil count were 35 and 1, respectively.

**FIGURE 2:**
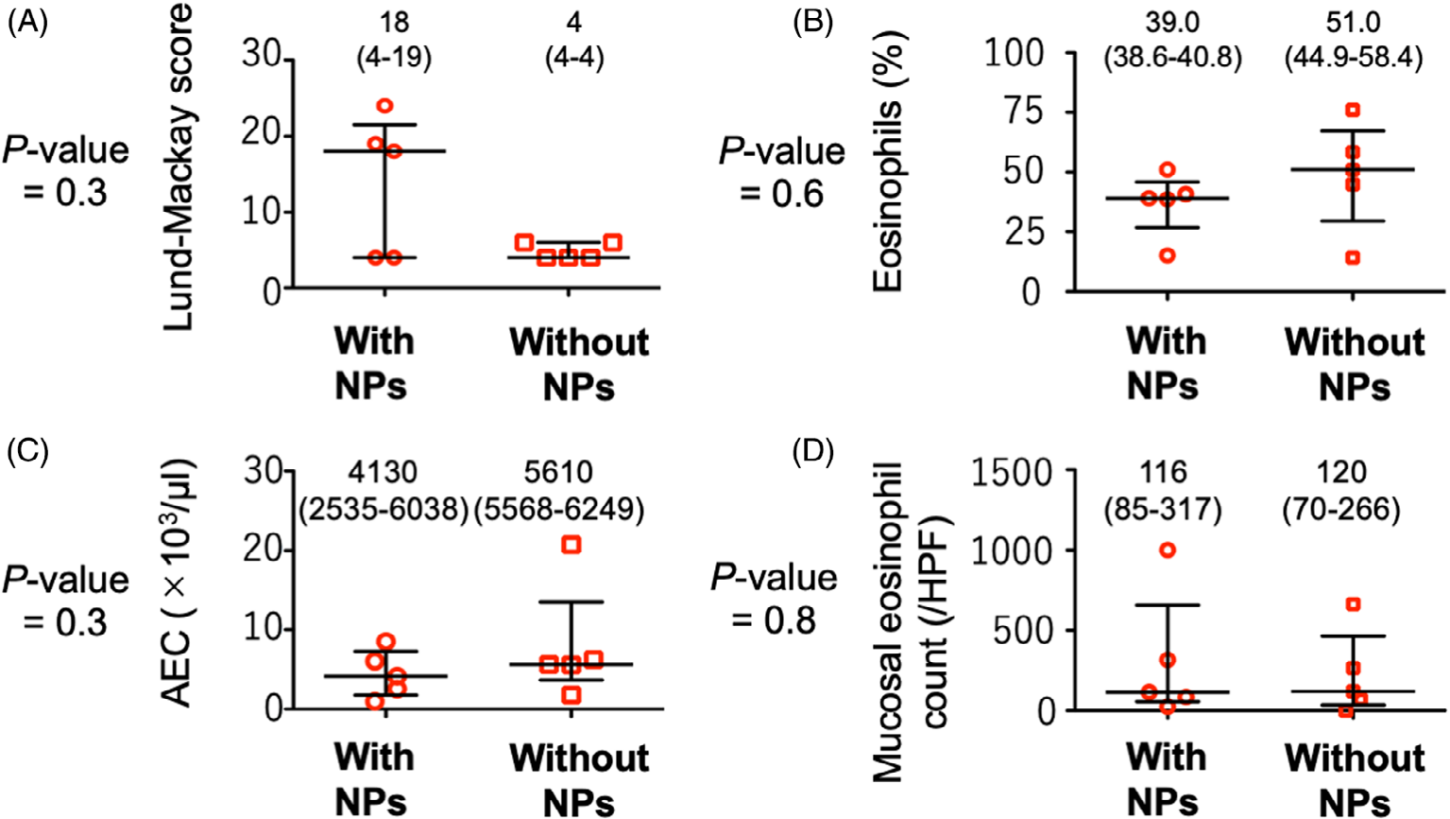
Comparison of Lund–Mackay CT total scores/eosinophilic data between comorbid CRS with and without NPs in CEP. The Mann–Whitney *U* test is used for the comparison: Lund–Mackay CT total score (A), percentage of eosinophils (B) and AEC (C) in blood, and mucosal eosinophil count (D). We set statistical significance at 5%. AEC, absolute eosinophil count; CEP, chronic eosinophilic pneumonia; CRS, chronic rhinosinusitis; CT, computed tomography; HPF, high‐power field; NPs, nasal polyps.

We investigated whether the Lund‐Mackay CT total scores/the eosinophilic infiltration in blood and sinonasal tissues can differ between CRS without and with NPs. In the Lund‐Mackay CT score, there was no significant difference between them (Figure 2A). However, the higher among‐individual variability was observed in CRS with NPs, compared to CRS without NPs. As shown in Figure 2B–D, both of CRS with and without NPs showed the median values of blood eosinophils percentage >35%, AEC > 4000/μL, and mucosal eosinophil count >110/HPF. In the statistical analyses targeting these data, there were no significant differences between CRS with and without NPs.

## DISCUSSION

Generally, CRS with NPs is a representative phenotype with high rates of relapse and comorbid asthma and is characterized by type 2‐related eosinophilic infiltration in sinonasal tissues.^4^ Although the presence of NPs can be useful in diagnosing CRS, our current results indicated the existence of CRS without NPs at the CEP onset. When pulmonologists have the opportunity to diagnose CEP, CT imaging not only of the chest but also of the sinonasal region may be effective in detecting comorbid CRS. In addition, otolaryngologists should not diagnose comorbid CRS based on endoscopic findings alone. The Lund‐Mackay CT score ranged from 4 to 24, meaning the considerable among‐individual variability in CT findings of paranasal sinus lesions.

Blood and sinonasal mucosa eosinophilia was one of the remarkable findings about comorbidity of CRS in CEP independent to the existence of NPs (see Figure 2B–D). For otolaryngologists, the presence of NPs is one of crucial factors to suspect eosinophilic CRS.^8^ As the eosinophilic CRS with NPs can show less treatment outcomes in comparison to the non‐eosinophilic one, the eosinophilic CRS diagnosis is important for otolaryngologists.^8^ However, some studies reported the existence of eosinophilic CRS without NPs, which showed poor treatment outcomes and high postoperative recurrence.^9^, ^10^, ^11^ One recent study reported increased eosinophilic inflammation in blood and sinonasal samples obtained from type 2 CRS without NP.^10^ In the JESREC scoring system,^7^ the existence of NPs is not absolutely essential for eosinophilic CRS diagnosis. In our study, CRS without NPs showed total scores ≥11 due to only two factors (the disease side and peripheral eosinophilic percentage). Considering the higher weight of blood eosinophilic percentage rather than the other factors in the JESREC scoring system, the eosinophilic data obtained from blood test might be useful to predict eosinophilic CRS without NPs. For the eosinophilic CRS diagnosis, the mucosa eosinophil count is essential,^8^ but there have been no global consensus of the cutoff values defining eosinophilic CRS. The European Position Paper defined eosinophilic CRS as an eosinophil count in sinonasal tissues of ≥10/HPF,^4^ and 90% of patients in the current study qualified for the diagnosis of eosinophilic CRS. Although the criteria for eosinophilic CRS in the JESREC scoring system require more conditions to be fulfilled, 80% of patients qualified as having eosinophilic CRS.

The considerable among‐individual variability observed in sinonasal CT findings was interesting. One recent study targeting EGPA investigated sinonasal CT findings, and then revealed in the following: (A) The Lund‐Mackay CT total score in EGPA can be lower than that in NSAID‐exacerbated respiratory disease and eosinophilic CRS without asthma; (B) EGPA cases with low Lund‐Mackay CT scores can represent only minor inflammation in anterior ethmoid and maxillary sinus, while high ones can represent severe inflammation in ostiomeatal complex; (C) EGPA cases with low Lund‐Mackay CT scores showed the higher frequency of extra‐respiratory organ involvement.^12^ The authors indicated that the sinonasal CT findings might reflect partial heterogeneity of pathological background in EGPA. As we did not focus on CRS without CEP in our current study, it still remains unclear whether the sinonasal CT findings can be different between CRS without and with CEP. The findings of (B) can be similar to those observed in our current study. CEP can be observed in the eosinophilic phase of EGPA, being the major feature usually preceding the symptoms of vasculitis.^13^ As well as CT findings in EGPA, the sinonasal CT might reflect the heterogeneity of pathological background in CEP. Further study should investigate whether the among‐individual variability in the sinonasal CT findings can possess clinical meaning such as the different clinical outcomes.

In conclusion, eosinophilic infiltration can occur simultaneously in the blood, sinonasal and lung tissues. The eosinophilic CRS can be a comorbidity in patients with CEP. For the diagnosis, sinonasal CT, biopsy, and eosinophilic data in blood tests can be crucial for diagnosis under the collaboration between the otolaryngologists and pulmonologists, regardless of the presence of NPs.

## AUTHOR CONTRIBUTIONS

*Concept and design*: Masaaki Ishikawa. *Drafting of the manuscript*: Masaaki Ishikawa and Hiroatsu Hatsukawa. *Critical revision of the manuscript for important intellectual content*: Masaaki Ishikawa. *Statistical analysis*: Masaaki Ishikawa and Hiroatsu Hatsukawa. *Administrative, technical, or material support*: Tomoyuki Hirai, Kazuo Endo, Emiko Saito, Hirotaka Matsumoto, Kouya Okazaki.

## CONFLICT OF INTEREST STATEMENT

None declared.

## ETHICS STATEMENT

The Research Ethics Committee in our institution approved the study protocol (approval no. 3–18). Informed consent was obtained from all patients to obtain sinonasal samples and explore the CRS phenotypes.

## Data Availability

Data supporting the findings of this study are available upon request from the corresponding authors.
